# Pioglitazone, bladder cancer, and detection bias

**DOI:** 10.1111/1753-0407.12087

**Published:** 2013-10-31

**Authors:** Paul Dolin

**Affiliations:** Takeda Development Centre EuropeLondon, UK

In their review of diabetes and cancer, Wang et al.^1^ have brought to our attention the 8-year interim analysis of the Kaiser Permanente Northern California (KPNC) cohort study of pioglitazone and bladder cancer,^2^ and the finding that adjusting for albuminuria attenuated the risk estimates. New additional analyses from the KPNC investigators have been posted to the European Medicines Agency's European Network of Centres for Pharmacoepidemiology and Pharmacovigilance (ENCePP) website and indicate the association may have been due to detection bias.^3^ It was found that there was more urinary monitoring for albuminuria among pioglitazone-treated patients, resulting in increased detection of subclinical hematuria and leading to a detection bias for bladder cancer. Adjusting for monitoring for albuminuria shifted the KPNC risk estimate to the null. No previous observational studies of pioglitazone had considered this detection bias. A recent Canadian cohort study has similarly suggested that an increased frequency of physician visits (e.g. to intensify monitoring or switch diabetic medications) may lead to the discovery of undiagnosed bladder cancer and result in a detection bias for bladder cancer in observational studies of diabetic patients.^4^

One should then question the recent report from an International Agency for Research on Cancer (IARC) Working Group that classified pioglitazone was “probably carcinogenic in humans”.^5^ This classification was based on an IARC algorithm, and caution is needed when interpreting their assessment. The first part of the algorithm was based on human data. The IARC group concluded there was “limited evidence of carcinogenicity in humans”. This IARC phrase is used when an association has been observed but a causal association has not been established, and the role of bias and confounding cannot be ruled out. The IARC group had full access to the KPNC 8-year interim analysis study reports, but unfortunately omitted these from their assessment.

The second part of the IARC algorithm was based on animal data. The IARC group concluded there was “sufficient evidence of carcinogenicity in animals”. Bladder neoplasms occurred in two long-term studies in male rats, but not in female rats and not in other species.^5^ The bladder tumors in male rats were considered to result from a proliferative response to chronic irritation following formation and retention of microcrystals. The tumors were specific to the ventral surface of the rat bladder, an area of prediction for deposition of solids in horizontal quadrupeds. Mechanistic studies acidifying the urine in male rats largely prevented the formation of bladder tumors, suggesting that the tumors were due to the formation of microcrystals in male rats.

However, this mode of action has little relevance to humans, a point the IARC group appears not to have fully considered. The osmolarity, pH and protein concentration of human urine differ to those in rats, and are far less favorable for microcrystal formation. At a previous IARC meeting it was concluded that this mode of action for the formation of bladder tumors in male rats is probably species specific and of limited relevance to humans.^6^ In conclusion, the IARC Group categorization of pioglitazone as “probably carcinogenic in humans” was not based on all available human data, and on animal and mechanistic data, the relevance of which to humans is questionable.

Pioglitazone plays a beneficial role in the treatment of diabetes. The US Food and Drug Administration and other health agencies have reviewed all available data and concluded that pioglitazone has a positive benefit risk ratio (see http://www.ema.europa.eu/ema/index.jsp?curl=pages/news_and_events/news/2011/10/news_detail_001368.jsp&mid=WC0b01ac058004d5c1 and http://www.fda.gov/Drugs/DrugSafety/ucm266555.htm, accessed 6 September 2013). The dubious conclusions of the IARC group will unfortunately have a negative impact on patient care worldwide.

## Disclosure

The author is employed by Takeda Development Centre (Europe) as Head of Pharmacoepidemiology. Takeda manufactures and markets pioglitazone. The author attended the IARC Monograph Volume 108 (Some drugs and herbal products) Working Group as an Observer.
